# Combined application of mesenchymal stem cells and different glucocorticoid dosing alleviates osteoporosis in SLE murine models

**DOI:** 10.1002/iid3.1319

**Published:** 2024-06-18

**Authors:** Sisi Ding, Tian Ren, Saizhe Song, Cheng Peng, Cuiping Liu, Jian Wu, Xin Chang

**Affiliations:** ^1^ Jiangsu Institute of Clinical Immunology The First Affiliated Hospital of Soochow University Suzhou China; ^2^ Department of Rheumatology The First Affiliated Hospital of Soochow University Suzhou China

**Keywords:** bone mesenchymal stem cells, glucocorticoids, osteoporosis, systemic lupus erythematosus

## Abstract

**Objective:**

Bone mesenchymal stem cells (BMSCs) have been tentatively applied in the treatment of glucocorticoid‐induced osteoporosis (GIOP) and systemic lupus erythematosus (SLE). However, the effects of BMSCs on osteoporosis within the context of glucocorticoid (GC) application in SLE remain unclear. Our aim was to explore the roles of BMSCs and different doses of GC interventions on osteoporosis in SLE murine models.

**Methods:**

MRL/MpJ‐Faslpr mice were divided into eight groups with BMSC treatment and different dose of GC intervention. Three‐dimensional imaging analysis and hematoxylin and eosin (H&E) staining were performed to observe morphological changes. The concentrations of osteoprotegerin (OPG) and receptor activator of nuclear factor κB ligand (RANKL) in serum were measured by enzyme‐linked immunosorbent assay (ELISA). The subpopulation of B cells and T cells in bone marrows and spleens were analyzed by flow cytometry. Serum cytokines and chemokines were assessed using Luminex magnetic bead technology.

**Results:**

BMSCs ameliorated osteoporosis in murine SLE models by enhancing bone mass, improving bone structure, and promoting bone formation through increased bone mineral content and optimization of trabecular morphology. BMSC and GC treatments reduced the number of B cells in bone marrows, but the effect was not significant in spleens. BMSCs significantly promoted the expression of IL‐10 while reducing IL‐18. Moreover, BMSCs exert immunomodulatory effects by reducing Th17 expression and rectifying the Th17/Treg imbalance.

**Conclusion:**

BMSCs effectively alleviate osteoporosis induced by SLE itself, as well as osteoporosis resulting from SLE combined with various doses of GC therapy. The therapeutic effects of BMSCs appear to be mediated by their influence on bone marrow B cells, T cell subsets, and associated cytokines. High‐dose GC treatment exerts a potent anti‐inflammatory effect but may hinder the immunotherapeutic potential of BMSCs. Our research may offer valuable guidance to clinicians regarding the use of BMSC treatment in SLE and provide insights into the judicious use of GCs in clinical practice.

## Graphical abstract

We investigated the effects of bone mesenchymal stem cells (BMSCs) and different doses of glucocorticoid (GC) interventions on osteoporosis in systemic lupus erythematosus (SLE) murine models. Our resultsdemonstrated that BMSCs could effectively alleviate osteoporosis induced by SLE itself, as well as osteoporosis resulting from SLE combined with various doses of GC therapy. These findings may offer valuable guidance to clinicians regarding the use of BMSC treatment in SLE and provide insights into the judicious use of GCs in clinical practice.

## 1 INTRODUCTION

Osteoporosis (OP) is a prevalent systemic bone disorder characterized by reduced bone mass and structural deterioration of bone tissue.^1^, ^2^, ^3^ It can be classified into primary osteoporosis and secondary osteoporosis. Secondary osteoporosis arises from various conditions or medications that impact bone metabolism, along with other well‐defined underlying causes.^4^, ^5^, ^6^ Currently, glucocorticoid‐induced osteoporosis (GIOP) is the most prevalent form of secondary osteoporosis.^7^ Glucocorticoids (GCs) find applications in clinical management of numerous ailments, with systemic lupus erythematosus (SLE) being one of the most common and representative instances.^8^, ^9^ The pathogenesis of SLE involves multiple immune cells and cytokines, yielding a complex immunopathological mechanism that companined with severe osteoporosis.^10^, ^11^ A meta‐analysis involving 33,527 SLE patients revealed that 45% of them experience reduced bone density, with 13% developing osteoporosis.^12^ Another study focusing on SLE patients in southern China found that only 7% had normal bone mass.^13^ Importantly, the emergence of osteoporosis in SLE is not solely attributed to GC usage; rather, the immune status intrinsic to the disease exerts an impact on cells and cytokines pertinent to bone metabolism. Despite this, previous studies have predominantly concentrated on the effects of GC treatment dosage and duration on GIOP occurrence, ignoring the interplay between immune status and GC application on bone metabolism. Recent years have brought forth the field of osteoimmunology, revealing that bone cells and immune cells share a common microenvironment within the bone marrow, characterized by the exchange of a multitude of cytokines, receptors, signaling molecules, and transcription factors.^14^, ^15^, ^16^ The interaction between the immune status of SLE and bone metabolism, along with the impact of GCs on both the immune system and bone metabolism, renders the emergence of osteoporosis within SLE patients more intricate than in other diseases. Consequently, to investigate the immune status may contribute to our comprehension of the pathological milieu characterizing this disease.

Mesenchymal stem cells (MSCs) are somatic stem cells endowed with the ability to self‐renew and undergo multidirectional differentiation.^17^ Since relatively easy to obtain and culture, MSCs have been tentatively applied for treating various diseases in recent years.^18^, ^19^ A study reported that systemic transplantation of human bone marrow MSCs and stem cells from exfoliated deciduous teeth could ameliorate severe bone reduction, as well as primary SLE disorders, in MRL/lpr mice.^20^ However, the impact of MSCs on osteoporosis within the context of GC application in SLE and the precise underlying mechanism remain to be elucidated. Our objective is to investigate the roles and potential immune mechanisms of MSCs in osteoporosis associated with SLE, across different dosages of GC administration. Moreover, attaining a more profound comprehension of these intricate disease states could pave the way for identifying more suitable therapeutic interventions.

## 2 MATERIALS AND METHODS

### 2.1 Mice

C57/BL6J mice (male, 10 weeks old) were purchased from Shanghai Sipper‐BK laboratory animal Company. MRL/MpJ‐Faslpr mice (male, 7‐8 weeks old) were purchased from Shanghai Jihui Laboratory Animal Care Co. Ltd. All employed mice were maintained in a specific pathogen‐free environment, residing within a controlled setting of consistent temperature and humidity. The animal studies conducted received ethical approval from the Institutional Animal Care and Use Committee of the First Affiliated Hospital of Soochow University.

### 2.2 Reagents and antibodies

Phorbol 12‐myristate 13‐acetate (PMA) and ionomycin were purchased from Sigma‐Aldrich. Fluorochrome‐conjugated anti‐mouse monoclonal antibodies (mAbs) including CD3 (clone: 145‐2C11), CD8 (clone: 6D5), B220 (clone: RA3‐6B2), CD138 (clone: 281‐2), CD25 (clone: 3C7), FOXP3 (clone: 150D), CXCR5 (clone: L138D7), IFN‐γ (clone: XMG1.2), TNF‐α (clone: MP6‐XT22), IL‐4 (clone: 11B11), IL‐9 (clone: RM94A), IL‐17 (clone: TC11‐18H10.1) and IL‐22 (clone: poly5164) were from Biolegend. Fluorochrome‐conjugated anti‐mouse CD4 (clone: GK1.5), CD38 (clone: 90), CD62L (clone: MEL‐14), IL‐6 (clone: MP5‐20F3), IL‐21 (clone: FFA21) were from eBbioscience.

### 2.3 Isolation and culture of bone MSCs (BMSCs)

BMSCs were isolated from bone marrows of C57/BL6J mice using established methods. In brief, bone marrow was flushed from the tibiae and femurs of the mice and subsequently seeded at a density of 3 × 10^6^/mL in cell culture dishes (Corning). The cells were cultured in Dulbecco's Modified Eagles Medium (DMEM) with high glucose, supplemented with 10% fetal bovine sera. The medium was replaced every 2−3 days, and cells within the 3rd−5th passages were selected for subsequent experiments.

### 2.4 Intervention or treatment of mice

MRL/MpJ‐Faslpr mice were randomly divided into eight distinct groups as follows: (1) SLE control group (SLE‐C); (2) BMSC treatment group (BMSC), (3) low‐dose (L‐D) dexamethasone (13.5 mg/kg) treatment group (L‐D); (4) moderate‐dose (M‐D) dexamethasone (22.6 mg/kg) treatment group (M‐D); (5) high‐dose (H‐D) dexamethasone (45.1 mg/kg) treatment group (H‐D); (6) L‐D dexamethasone combined with BMSC treatment group (L‐D + B); (7) M‐D dexamethasone combined with BMSC treatment group (M‐D + B); (8) H‐D dexamethasone combined with BMSC treatment group (H‐D + B). For BMSC treatment groups, intraperitoneal (ip) injections of BMSCs (2 × 10^6^ cells/mouse) were administered every 2 weeks. Diverse doses of dexamethasone were also administered via ip injections weekly for the DEX treatment group, categorized by the respective dosage. Each cycle consisted of 4 weeks of intervention or treatment and was maintained for a duration of 16 weeks. Following this period, the mice were humanely euthanized to obtain spleen, femurs, and tibias for subsequent experimental procedures.

### 2.5 Microcomputed tomography (CT) analysis

The paws and femurs of the mice were fixed in a 4% paraformaldehyde solution for 48 h. Subsequent examination of bone lesions and morphometric parameters was conducted utilizing micro‐CT (VENUS 001, PINGSENG Health care). The acquired data were reconstructed and subjected to analysis through the utilization of the Avatar 3 Visualization and Analysis Software.

### 2.6 ELISA

Blood samples were obtained through orbital puncture and serum was then separated by centrifugation. Enzyme‐linked immunosorbent assay (ELISA) kits (Proteintech) were employed for detecting receptor activator of nuclear factor κB ligand (RANKL) and osteoprotegerin (OPG) levels in serum derived from mice. The optical density (OD) values were read at 450 nm and quantified using an ELISA reader equipped with specialized software (Thermo).

### 2.7 Flow cytometry

Bone marrows extracted from femurs and spleens from mice were collected for flow cytometry analysis. Fluorochrome‐conjugated anti‐mouse mAbs were introduced to 100 µL of the single cell suspensions and were allowed to incubate for 30 min at 4°C. For intracellular staining, cells were initially stimulated with PMA (50 ng/mL) and ionomycin (1 µg/mL). Following fixation and permeabilization, fluorochrome‐conjugated mAbs targeting specific markers including IFN‐γ, TNF‐ɑ, IL‐4, IL‐6, IL‐9, IL‐17, IL‐21, and IL‐22 were added. The antigenic properties of the cells were analyzed using the COULTER Epics XL flow cytometer (Beckman Coulter).

### 2.8 Luminex magnetic bead technology

A ProcartaPlex Immunoassay Kit (Invitrogen, ThermoFisher Scientific) was used to conduct quantitative, multiplexed protein measurements on serum samples obtained from mice. The immunoassay was conducted according to manufacturers' instructions incorporated Luminex magnetic bead technology.

### 2.9 Statistical analysis

Statistical analysis was carried out by Microsoft Office Excel and Graphpad prism (Version 9.0). For independent samples, either Student's *t*‐test or the nonparametric Mann−Whitney *U* test was utilized, while paired samples underwent analysis through either the paired *t*‐test or the nonparametric Wilcoxon signed‐rank test. Multiple comparisons were assessed using either the one‐way analysis of variance or the Kruskal−Wallis test. A significance level of *p* < .05 was adopted as the threshold for statistical significance.

## 3 RESULTS

### 3.1 BMSCs improve osteoporosis in murine SLE models

**Figure 1:**
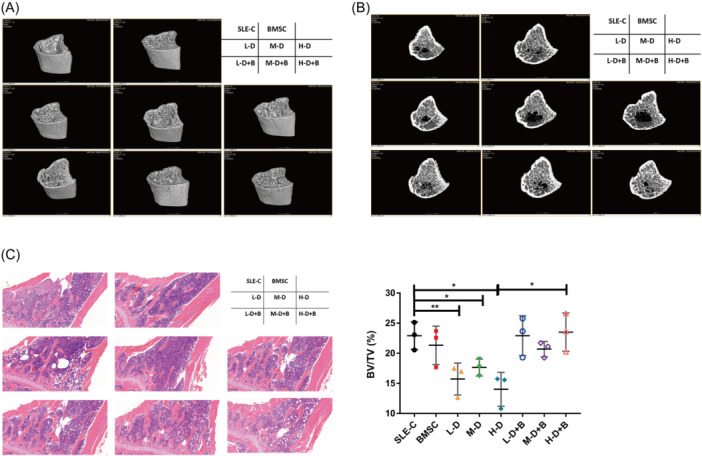
Three‐dimensional (3D) imaging and histology analysis on the left tibias of mice. (A) Representative micro‐CT images of longitudinal sections. (B) Representative micro‐CT images of transverse sections. (C) The representative images and quantification analysis of hematoxylin and eosin (H&E) staining. Magnification: ×5. CT, computed tomography.

Three‐dimensional (3D) imaging analysis was conducted on the left tibias of three mice within each group using micro‐CT. Compared with the SLE‐C group, the BMSC treatment group exhibited thickened cortical bone and increased mass of trabecular bones, along with a distinctly visible bone structure. As the dose of dexamethasone increased, 3D images depicted thinner and irregular cortical bone, accompanied by disorganized trabecular architecture and gradual density reduction (Figure 1A,B). The addition of BMSC treatment ameliorated the aforementioned trabecular bone abnormalities in all groups. To validate the radiological assessment by micro‐CT, hematoxylin and eosin (H&E) staining was performed to observe the morphological changes. Quantitation of bone volume relative to tissue volume (BV/TV, %) in diaphyseal regions was analyzed through Image J. While the levels of BV/TV were significantly decreased in Groups L‐D (*p* = .0082), M‐D (*p* = .0273) and H‐D (*p* = .0133), the trend reversed with combined BMSC treatment, which was particularly evident in the high‐dose dexamethasone treated group (*p* = .0181; Figure 1C). The results were consistent with micro‐CT findings, including alterations in trabecular bone number, density, and architecture.

**Figure 2:**
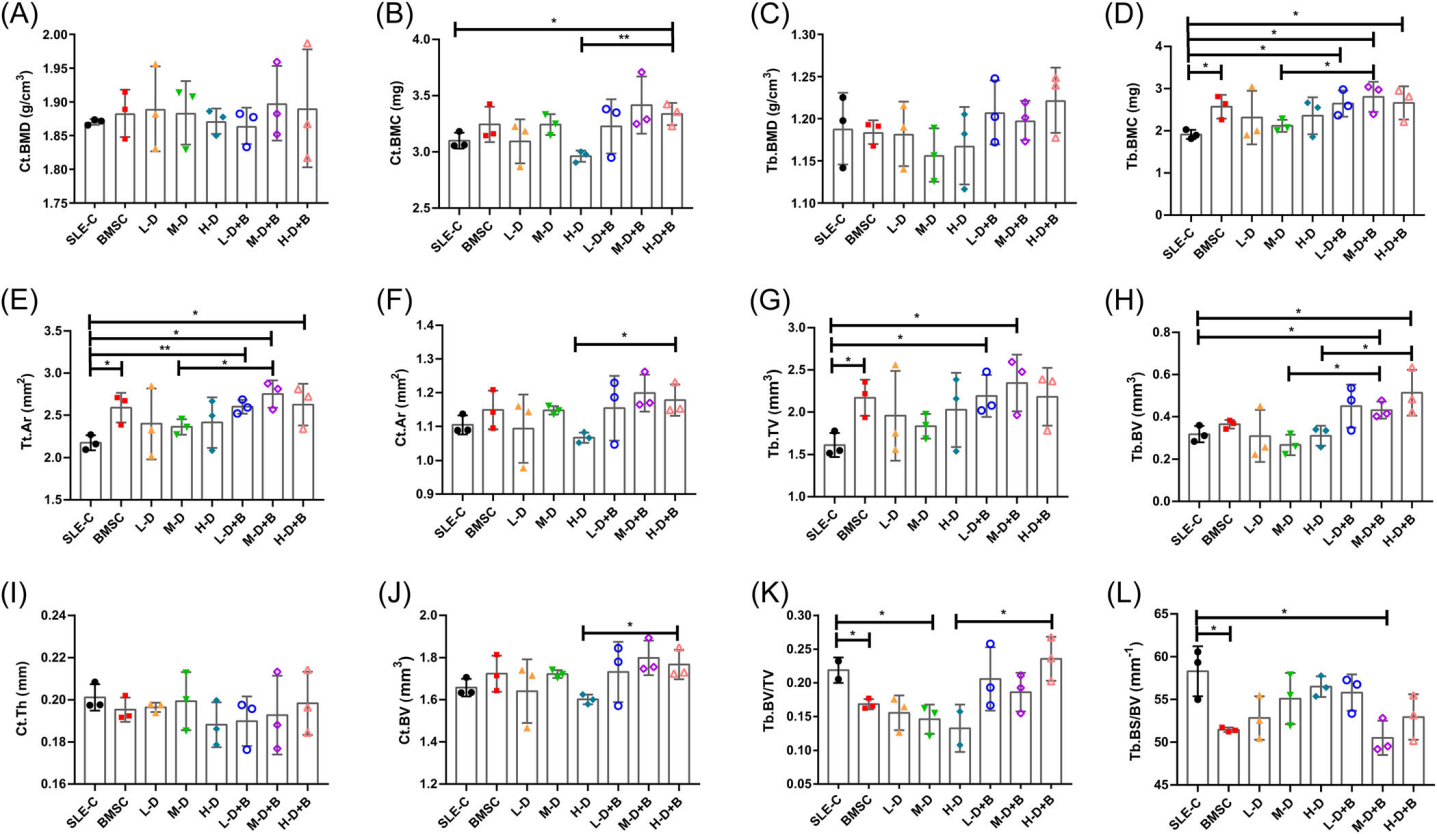
Analysis of bone microarchitecture parameters. (A) Bone mineral density of the cortical bone (Ct.BMD). (B) Bone mineral content of the cortical bone (Ct.BMC). (C) Bone mineral density of the trabecular bone (Tb.BMD). (D) Bone mineral content of the trabecular bone (Tb.BMC). (E) Cortical total area (Tt.Ar). (F) Cortical bone area (Ct.Ar). (G) Trabecular tissue volume (Tb.TV). (h) Trabecular bone volume (Tb.BV). (I) Cortical thickness (Ct.Th). (J) Cortical bone volume (Ct.BV). (K) Bone volume fraction of the trabecular bone (bone volume/tissue volume ratio, Tb.BV/TV). (L) Bone surface area/bone volume ratio of the trabecular bone (Tb.BS/BV). Bars indicate mean ± SD; *p < .05, **p < .01.

The micro‐CT data were then subjected to statistical analysis. Compared with the control, no significant variations were observed in cortical and trabecular bone mineral density (Ct.BMD and Tb. BMD) among different groups (Figure 2A,C). However, cortical bone mineral content (Ct.BMC) was significantly higher in H‐D + B group when compared to the SLE‐C group (3.33 ± 0.08 mg vs. 3.10 ± 0.06 mg, *p* = .0279; Figure 2B). When BMSC treatment was received, a significant higher level of Ct.BMC was observed in the H‐D dexamethasone treatment group (3.33 ± 0.08 mg vs. 2.96 ± 0.04 mg, *p* = .0042; Figure 2B). The levels of trabecular mineral content (Tb.BMC) were notably elevated in Groups BMSC (2.57 ± 0.23 mg, *p* = .0195), L‐D + B (2.65 ± 0.25 mg, *p* = .0179), M‐D + B (2.80 ± 0.29 mg, *p* = .0143), and H‐D + B (2.66 ± 0.32 mg, *p* = .0336; Figure 2D). Meanwhlie, a statistically significant difference was observed between M‐D + B and M‐D groups (2.80 ± 0.29 mg vs. 2.12 ± 0.12 mg, *p* = .0366; Figure 2D). The cortical total areas (Tt.Ar) were significantly increased in Groups BMSC (2.59 ± 0.14 mm^2^, *p* = .0228), L‐D + B (2.60 ± 0.06 mm^2^, *p* = .0038), M‐D + B (2.75 ± 0.13 mm^2^, *p* = .0057) and H‐D + B (2.63 ± 0.20 mm^2^, *p* = .0423) when compared to the SLE‐C group (2.18 ± 0.07 mm^2^; Figure 2E). We also found that the cortical bone area (Ct.Ar) was higher when combined with BMSC treatment in H‐D dexamethasone treated group (H‐D‐B vs. H‐D, 1.18 ± 0.04 mm^2^ vs. 1.07 ± 0.01 mm^2^, *p* = .0017; Figure 2F). Moreover, the trabecular tissue volumes (Tb.TV) were increased in Groups BMSC (2.17 ± 0.17 mm^3^, *p* = .0199), L‐D + B (2.19 ± 0.20 mm^3^, *p* = .0025) and M‐D + B (2.34 ± 0.27 mm^3^, *p* = .0025) when compared with SLE‐C group (Figure 2G). The value of trabecular bone volumes (Tb.BV) significantly increased in mice combined with BMSC treatment when compared with SLE‐C or corresponding moderate/high dose dexamethasone group (H‐D + B vs. H‐D, *p* = .0411; M‐D + B vs. M‐D, *p* = .0111; H‐D + B vs. SLE‐C, *p* = .0425; M‐D + B vs. SLE‐C, *p* = .0240; Figure 2H). Further analysis revealed that the trabecular bone volume fraction (bone volume/tissue volume ratio, Tb.BV/TV) exhibited significant decreases in Groups BMSC (0.17 ± 0.01, *p* = .0218) and M‐D (0.15 ± 0.02, *p* = .00319) when compared to the SLE‐C group (0.22 ± 0.01). This downward trend was reversed when combined with BMSC treatment, particularly evident in the H‐D dexamethasone treated group (H‐D‐B vs. H‐D, 0.24 ± 0.03 vs. 0.13 ± 0.02, *p* = .0434; Figure 2K). The trabecular bone surface area/bone volume ratios (Tb.BS/BV) were also significantly reduced in the BMSC (51.46 ± 0.22 mm^−1^, *p* = .0157) and M‐D + B treatment group (50.51 ± 1.64 mm^−1^, *p* = .0191; Figure 2L). Although there was no difference in cortical thickness (Ct.Th), the cortical bone volume (Ct.BV) significantly increased in H‐D dexamethasone treatment group (H‐D) when BMSC applied (*p* = .0175) (Figure 2I,J).

**Figure 3:**
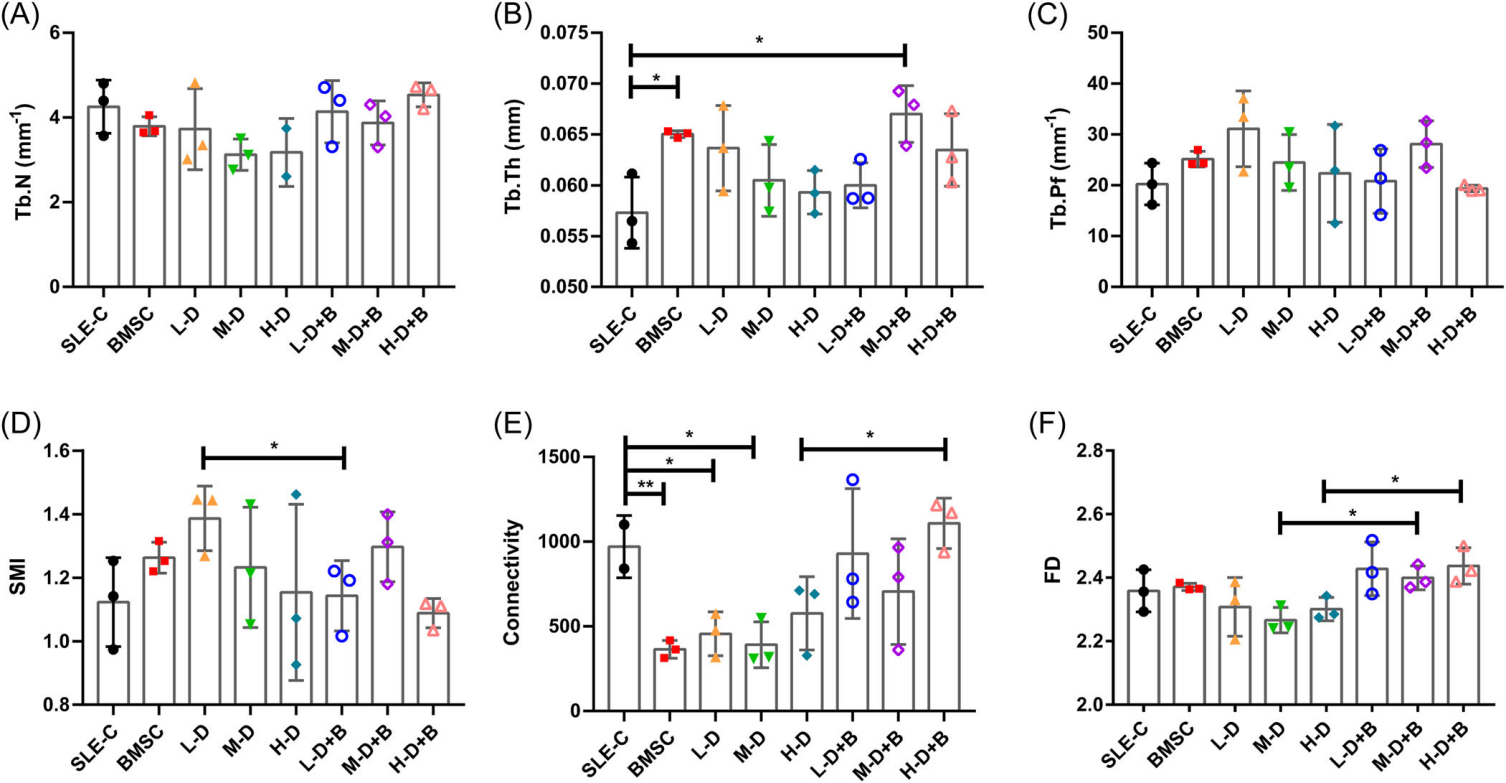
The parametric values of the trabecular bone microarchitecture. (A) Trabecular number (Tb.N). (B) Ttrabecular thickness (Tb.Th). (C) Trabecular pattern factor (Tb.Pf). (D) Structure mode index (SMI) of trabecular bone. (E) Connectivity of trabecular bone. (F) Fractal dimension (FD) of trabecular bone. Bars indicate mean ± SD; *p < .05, **p < .01.

In addition, we analyzed the parametric values of the trabecular bone microarchitecture including trabecular number (Tb.N), trabecular thickness (Tb.Th), trabecular pattern factor (Tb.Pf), structure mode index (SMI), connectivity and fractal dimension (FD). The levels of Tb.Th were significant higher in BMSC (*p* = .0189) and M‐D + B groups (*p* = .0197), while there were no differences in trabecular thickness or trabecular pattern factor (Figure 3A,B,C). Although there were no changes in SMI by treatment with either agent alone (BMSC or dexamethasone), the L‐D group displayed a significant improvement under BMSC treatment (1.14 ± 0.09 vs. 1.39 ± 0.08, *p* = .0483; Figure 3D). Each treatment group exhibited a decrease in trabecular connectivity compared to the control group, with statistical significant in Groups BMSC (363.33 ± 42.47, *p* = .0101), L‐D (456.00 ± 105.46, *p* = .0327), and M‐D (391.33 ± 110.86, *p* = .0256; Figure 3E). This tendency reversed with the addition of BMSC treatment in the H‐D group (1108.00 ± 121.54 vs. 577.00 ± 176.28, *p* = .0247; Figure 3E). The values of FD experienced significant increases in Groups H‐D + B and M‐D + B when compared to Groups H‐D and M‐D, respectively (H‐D + B vs. H‐D: 2.44 ± 0.05 vs. 2.30 ± 0.03, *p* = .0260; M‐D + B vs. M‐D: 2.40 ± 0.03 vs. 2.27 ± 0.03, *p* = .0137; Figure 3F).

**Figure 4:**
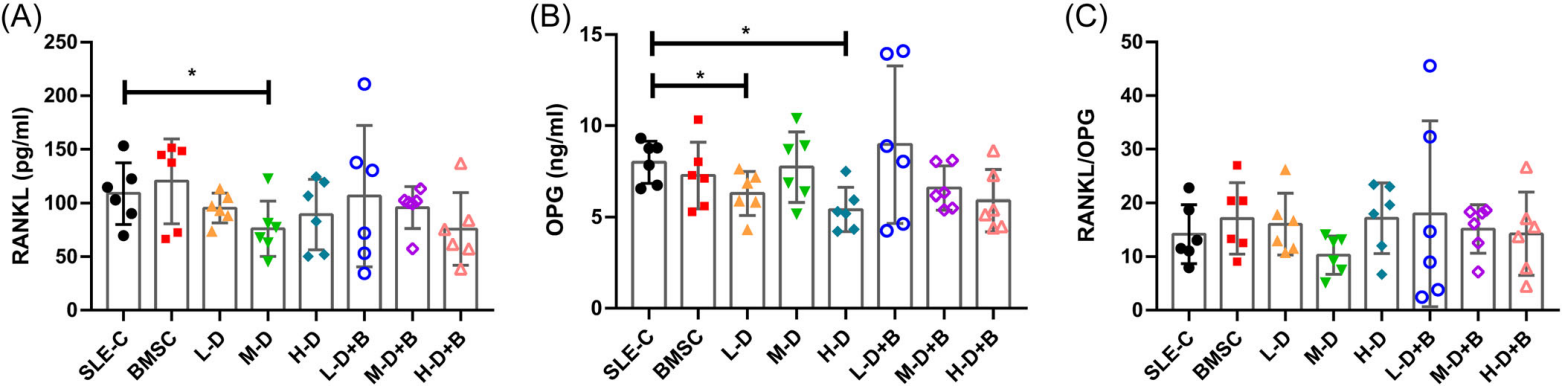
The concentrations of RANKL and OPG in serum of mice. (A) RANKL. (B) OPG. (C) OPG/RANKL ratio. Bars indicate mean ± SD; *p < .05.

Moreover, both H‐D and low‐dose (L‐D) dexamethasone treatments significantly downregulated serum levels of OPG (H‐D: 5.41 ± 1.10 ng/mL vs. 7.99 ± 1.05 ng/mL, *p* = .0036; L‐D: 6.29 ± 1.09 ng/mL vs. 7.99 ± 1.05 ng/mL, *p* = .0307; Figure 4B). Nonetheless, the concentrations of OPG and the RANKL/OPG ratio remained unchanged (Figure 4A,C). These findings collectively suggested that BMSCs hold the potential to ameliorate osteoporosis in murine SLE models.

### 3.2 Effects of BMSCs and different dose of GC intervention on B cell subsets

**Figure 5:**
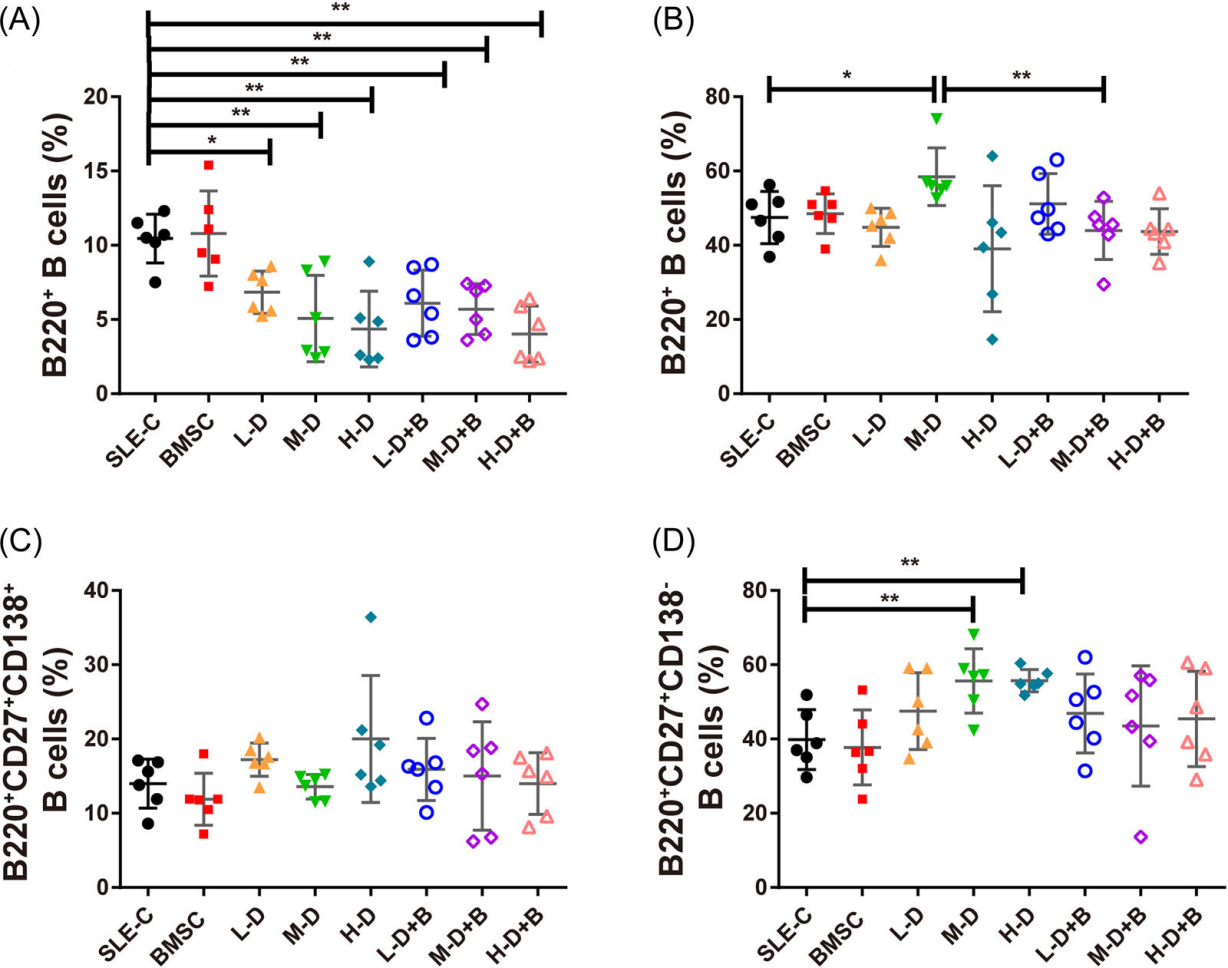
Effect of BMSCs and different dose of GC intervention on B cell subsets. (A) The percentages of B cells in the bone marrow of mice. (B) The percentages of B cells in the spleens of mice. (C) The percentages of plasmablast B cells (B220^+^CD27^+^CD138^+^) in the spleens of mice. (D) The percentages of memory B cells (B220^+^CD27^+^CD138^‐^) in the spleens of mice. Bars indicate mean ± SD; *p < .05, **p < .01, ***p < .001. BMSCs, bone mesenchymal stem cells; GC, glucocorticoid.

We first analyzed the percentages of B cell subsets in bone marrows and spleens in MRL/lpr mice. In bone marrows, each treatment group showed a significant downregulation in B cell population when compared with the controls (*p* < .01). However, no significant differences emerged between the GC treatment and the corresponding BMSC treatment groups *(p* > .05, Figure 5A). Moving on to spleens, we observed that the B cell population in the M‐D dexamethasone treatment group was elevated in contrast to the controls (58.45 ± 7.08% vs. 47.47 ± 6.42%, *p* = .0279). Yet, this population experienced a substantial reduction upon combination with BMSC treatment (43.98 ± 7.15% vs. 58.45 ± 7.08%, *p* = .0093; Figure 5B). B cells were then separated into memory B cells and plasmablast B cells. No significant differences were observed in plasmablast B cells (B220^+^CD27^+^CD138^+^) across the various groups (Figure 5C). In contrast, the percentages of memory B cells (B220^+^CD27^+^CD138^−^) were significantly augmented in Groups M‐D (55.62 ± 7.91%, *p* = .0085) and H‐D (55.70 ± 2.71%, *p* = .0011) (Figure 5D).

### 3.3 Effects of BMSCs and different dose of GC intervention on T cell subsets

**Figure 6:**
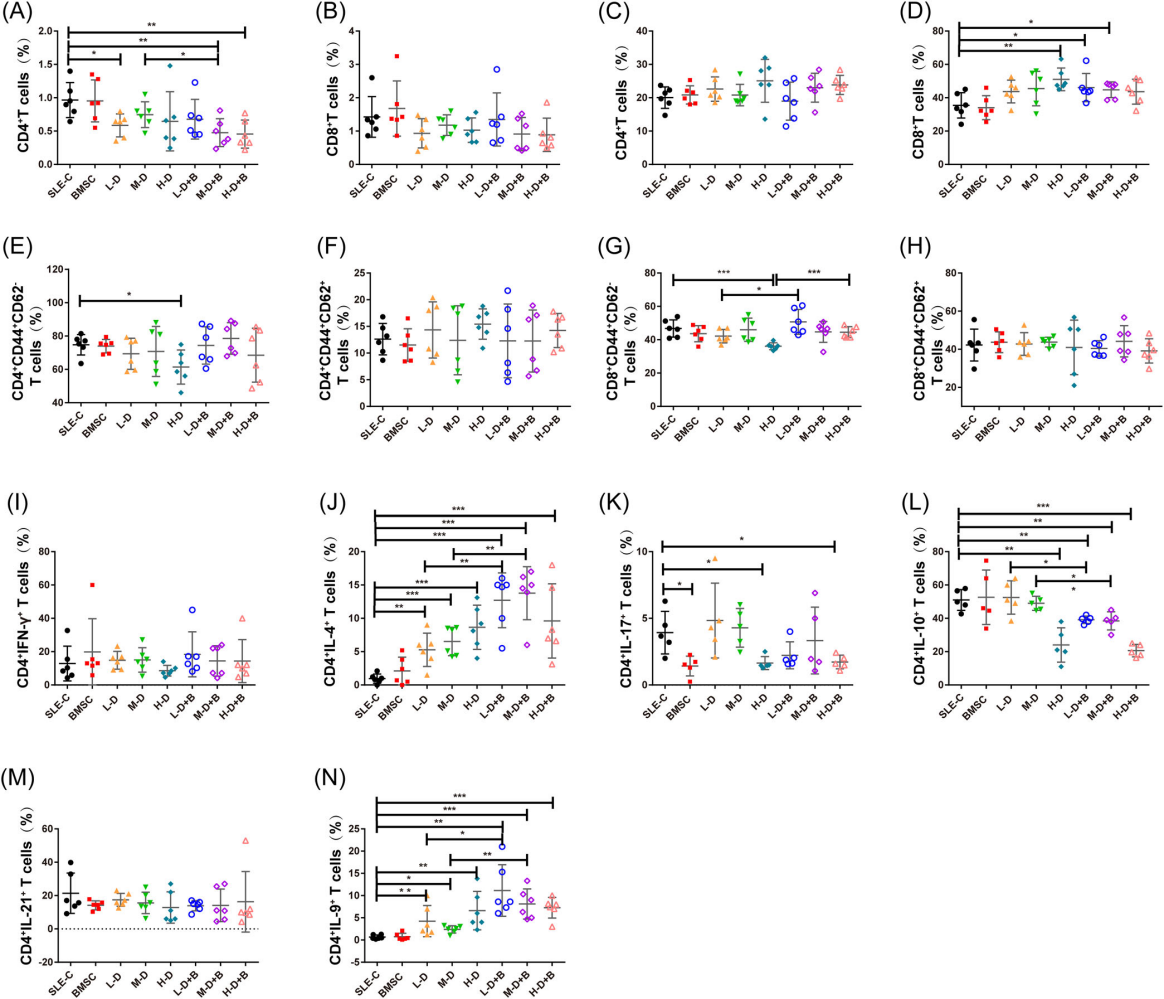
Effect of BMSCs and different dose of GC intervention on T cell subsets. (A) The percentages of CD4^+^ T cells in the bone marrow of mice. (B) The percentages of CD8^+^ T cells in the bone marrow of mice. (C) The percentages of CD4^+^ T cells in the spleens of mice. (D) The percentages of CD8^+^ T cells in the spleens of mice. (E) The percentages of CD4 effector T cells in mice. (F) The percentages of CD4 central memory T cells in mice. (G) The percentages of CD8 effector T cells in mice. (H) The percentages of CD8 central memory T cells in mice. (I−N) The percentages of Th1 cells (I), Th2 cells (J), Th17 cells (K), Tregs (L), Tfh cells (M) and Th9 cells (N) in mice. Bars indicate mean ± SD; *p < .05, **p < .01, ***p < .001. BMSCs, bone mesenchymal stem cells; GC, glucocorticoid.

In the bone marrows of mice, Groups L‐D (0.59 ± 0.16%, *p* = .0142), M‐D + B (0.48 ± 0.19%, *p* = .0051), and H‐D + B (0.46 ± 0.19%, *p* = .0041) showed significant reductions in CD4^+^ T cells compared to the SLE‐C group, and the reduction persisted as statistically significant between Groups M‐D + B and M‐D, which were subjected to BMSC treatment (0.48 ± 0.19% vs. 0.75 ± 0.18%, *p* = .0432; Figure 6A). There were no noteworthy differences in the proportions of CD8^+^ T cells (Figure 6B). In the spleens of mice, we observed distinct phenomena where CD8^+^ T cells were upregulated in three groups (Groups H‐D, L‐D + B, and M‐D + B) compared to controls (H‐D: 51.02 ± 5.76%, *p* = .0039; L‐D + B: 46.08 ± 7.17%, *p* = .0450; M‐D + B: 44.78 ± 3.97%, *p* = .0283), whereas there was no change in the number of spleen CD4^+^ T cells (Figure 6C,D). Compared with the SLE‐C control group, the population of CD4 and CD8 effector T cells experienced significant reductions in the H‐D group (CD4: 61.40 ± 8.62% vs. 74.77 ± 5.20%, *p* = .0205; CD8: 36.1 ± 1.75% vs. 46.75 ± 4.37%, *p* = .0009; Figure 6E,G). The CD8 effector T cells in Groups H‐D + B and L‐D + B were elevated in comparison to Group H‐D and L‐D respectively (H‐D + B vs. H‐D: 44.52 ± 2.79% vs. 36.10 ± 1.75%, *p* = .0004; L‐D + B vs. L‐D: 50.70 ± 6.33% vs. 42.13 ± 3.42%, *p* = .0334; Figure 6G). There were no significant differences in the proportions of CD4 or CD8 central memory T cells (Figure 6F,H). Additionally, we analyzed several CD4^+^ T cell subsets, including Th1, Th2, Th17, Treg, Tfh, and Th9 cells. No significant changes were noted in Th1 or Tfh populations (Figure 6I,M). While the amounts of Th2 and Th9 cells were not significantly altered in the BMSC treatment group compared to SLE‐C, they were significant enhanced in the remaining groups, with the increase being more pronounced upon combination with BMSC treatment (*p* < .05, Figure 6J,N). Conversely, the population of Treg cells underwent a substantial decrease (*p* < .05, Figure 6L). The numbers of Th17 cells were lower in Groups BMSC, H‐D, and H‐D + B than in controls (*p* < .05, Figure 6K).

### 3.4 Alterations in serum cytokines and chemokines in response to BMSC and GC intervention

**Figure 7:**
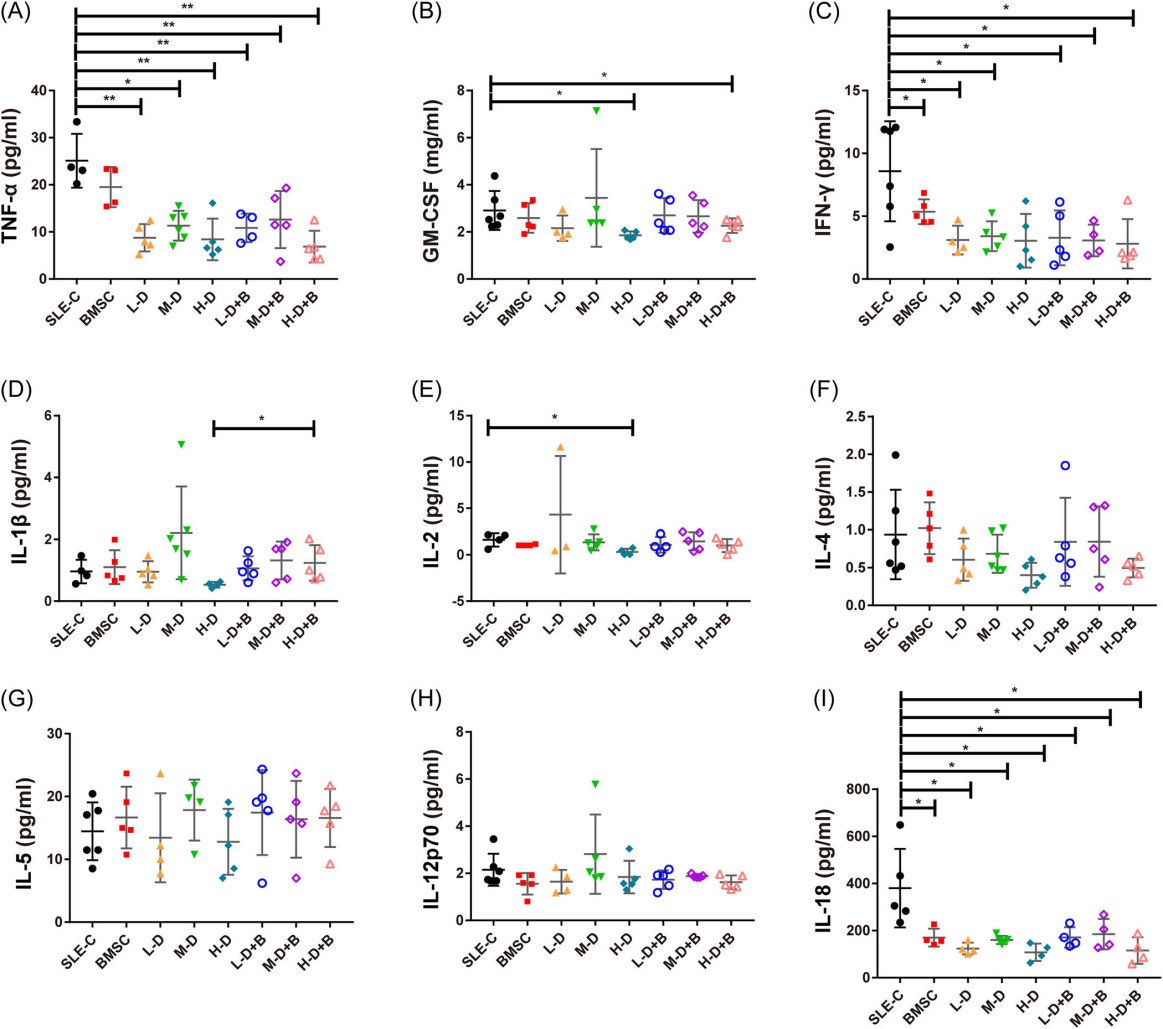
The expression of Th1/Th2 related cytokines in serum samples of mice. (A) The levels of TNF‐α in serum of mice. (B) The levels of GM‐SCF in serum of mice. (C) The levels of IFN‐γ in serum of mice. (D) The levels of IL‐1β in serum of mice. (E) The levels of IL‐2 in serum of mice. (F) The levels of IL‐4 in serum of mice. (G) The levels of IL‐5 in serum of mice. (H) The levels of IL‐12p70in serum of mice. (I) The levels of IL‐18 in serum of mice. Bars indicate mean ± SD; *p < .05, **p < .01.

**Figure 8:**
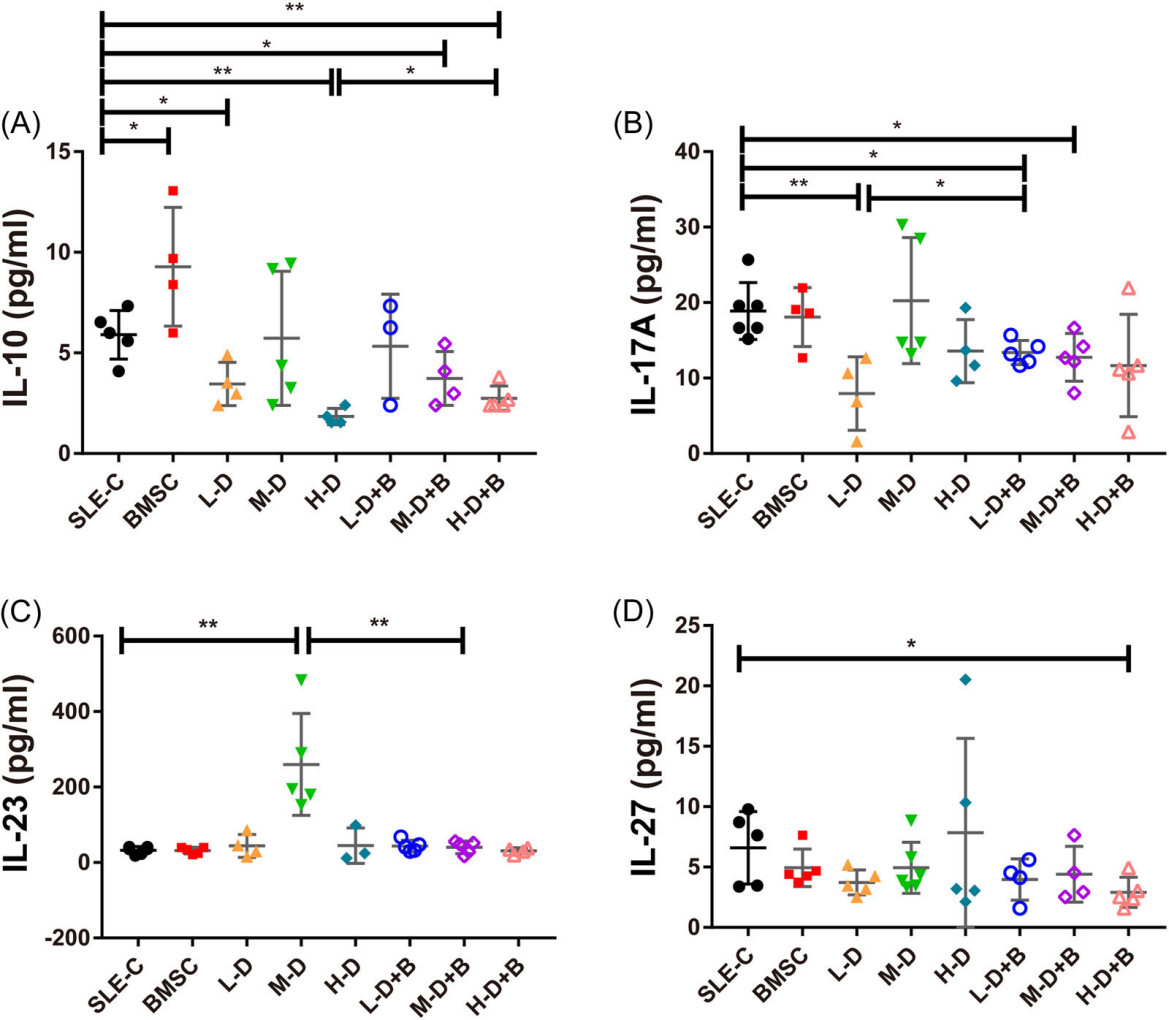
The expression of Th17/Treg related cytokines in serum samples of mice. (A) The levels of IL‐10 in serum of mice. (B) The levels of IL‐17A in serum of mice. (C) The levels of IL‐23 in serum of mice. (D) The levels of IL‐27 in serum of mice. Bars indicate mean ± SD; *p < .05, **p < .01.

**Figure 9:**
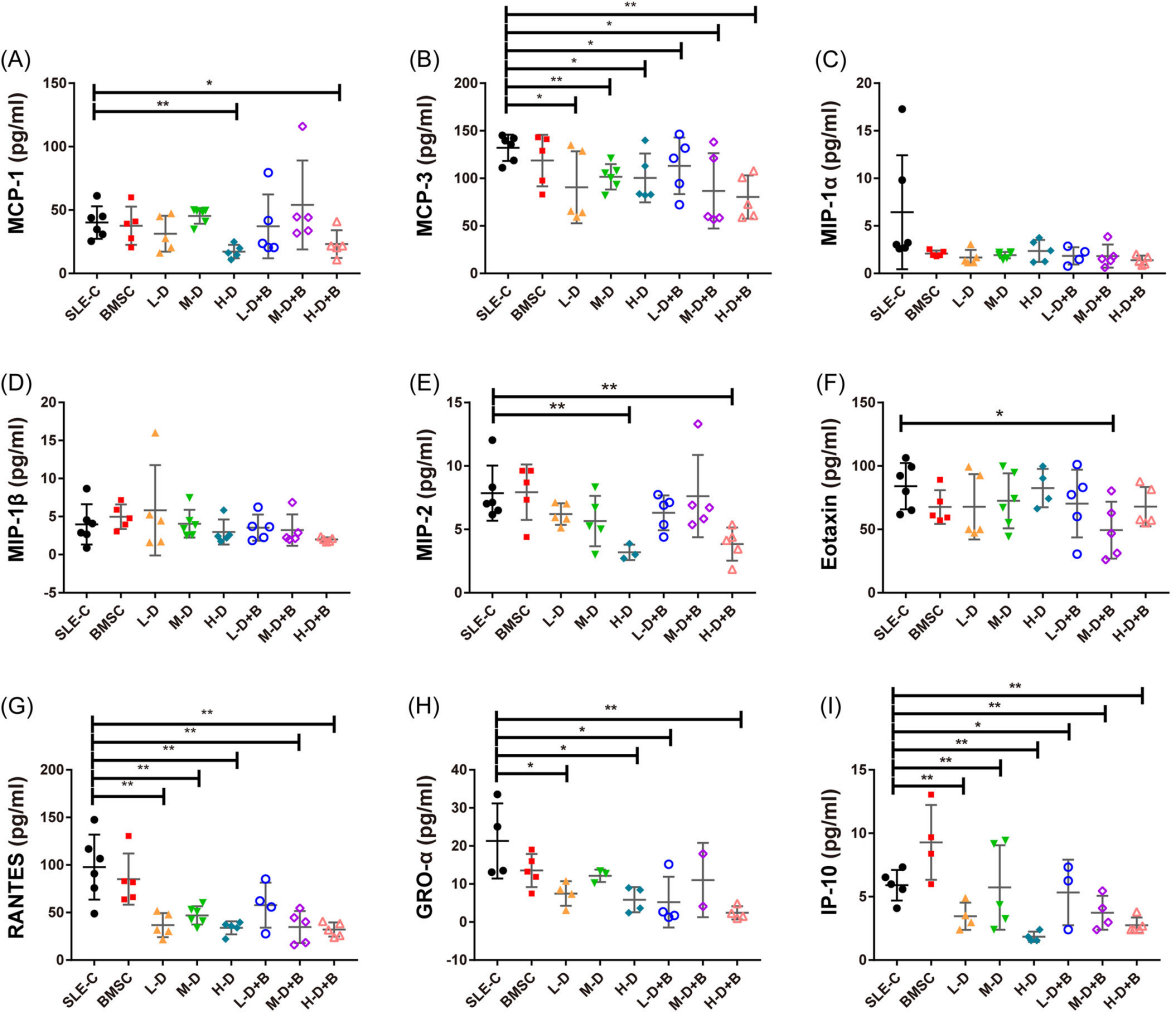
The expression of several chemokines in serum samples of mice. (A) The levels of MCP‐1 in serum of mice. (B) The levels of MCP‐3 in serum of mice. (C) The levels of MIP‐1α in serum of mice. (D) The levels of MIP‐1β in serum of mice. (E) The levels of MIP‐2 in serum of mice. (F) The levels of eotaxin in serum of mice. (G) The levels of RANTES in serum of mice. (H) The levels of GRO‐α in serum of mice. (I) The levels of IP‐10 in serum of mice. Bars indicate mean ± SD; *p < .05, **p < .01. MCP, monocyte chemoattractant protein; MIP, macrophage inflammmatory protein.

To evaluate the potential influence of BMSC and GC treatments on the inflammatory microenvironment, we investigated the expression of several chemokines, and cytokines related to Th1/Th2 and Th17/Treg pathways in serum samples of mice by Luminex magnetic bead technology. Our analysis encompassed nine Th1/Th2 related cytokines, four Th17/Treg related cytokines, and nine chemokines. Detailed values are shown in Tables 1, 2, 3. Among the nine Th1/Th2 related cytokines, five of them (TNF‐α, GM‐CSF, IFN‐γ, IL‐2, and IL‐18) exhibited significant reductions across various treated groups, while other chemokines displayed minimal or no discernible alterations (*p* < .05, Figure 7). Within the Th17/Treg related cytokines, IL‐10 and IL‐17A demonstrated various degrees of downregulation in both BMSC and GC treatment groups in comparison to controls. Notably, this trend was reversed within the low or H‐D dexamethasone treatment groups upon the addition of BMSCs (*p* < .05, Figure 8). Regarding chemokines, monocyte chemoattractant protein (MCP‐1), MCP‐3, macrophage inflammmatory protein (MIP‐2), eotaxin, RANTES, GRO‐α, and IP‐10 were markedly diminished in the BMSC and GC treatment groups compared to the SLE‐C group. However, no distinctions were observed in GC combined with BMSC treatment groups when contrasted to the corresponding GC treatment groups (*p* < .05, Figure 9).

**Table 1:** The levels of Th1/Th2 related cytokines in mice.

	SLE‐C	BMSC	D	D	D	L‐D + B	D + B	H‐D + B
TNF‐α	20.38 ± 7.99	21.06 ± 4.52	8.76 ± 2.60	11.31 ± 2.89	8.42 ± 3.95	14.65 ± 7.94	12.61 ± 5.42	6.87 ± 3.03
GM‐SCF	2.91 ± 0.75	2.59 ± 0.56	9.08 ± 13.85	5.92 ± 5.79	1.85 ± 0.15	2.7 ± 0.66	2.66 ± 0.62	2.27 ± 0.28
IFN‐γ	8.57 ± 3.63	5.36 ± 0.88	16.22 ± 26.26	5.52 ± 4.83	3.04 ± 1.91	3.28 ± 1.95	4.26 ± 2.57	2.8 ± 1.75
IL‐1β	1.03 ± 0.49	1.10 ± 0.49	0.95 ± 0.31	2.21 ± 1.37	0.87 ± 0.69	1.06 ± 0.35	1.32 ± 0.55	1.24 ± 0.51
IL‐2	3.14 ± 3.10	1.99 ± 1.88	4.32 ± 5.16	5.25 ± 8.51	0.32 ± 0.30	1.08 ± 0.73	1.46 ± 0.87	1.00 ± 0.63
IL‐4	0.94 ± 0.54	1.02 ± 0.31	0.61 ± 0.25	0.68 ± 0.23	0.40 ± 0.15	0.84 ± 0.52	0.84 ± 0.41	0.50 ± 0.11
IL‐5	14.46 ± 4.20	16.65 ± 4.40	23.2 ± 20.32	25.14 ± 14.42	12.79 ± 4.69	17.44 ± 6.03	16.38 ± 5.47	16.59 ± 4.13
IL‐12p70	2.15 ± 0.62	1.55 ± 0.41	12.93 ± 22.57	6.32 ± 7.96	1.84 ± 0.62	1.73 ± 0.35	1.88 ± 0.06	1.62 ± 0.26
IL‐18	336.76 ± 167.53	196.84 ± 60.54	568.05 ± 888.44	549.21 ± 869.44	107.90 ± 32.00	139.38 ± 71.37	254.28 ± 146.84	99.56 ± 53.72

Abbreviations: BMSCs, bone mesenchymal stem cells; GC, glucocorticoid; H‐D, high‐dose; L‐D, low‐dose; M‐D, moderate‐dose; SLE‐C, systemic lupus erythematosus control.

**Table 2:** The levels of Th17/Treg related cytokines in mice.

	SLE‐C	BMSC	L‐D	M‐D	L‐D	L‐D + B	M‐D + B	H‐D + B
IL‐10	6.65 ± 2.64	8.25 ± 4.36	5.96 ± 4.24	4.88 ± 3.18	3.20 ± 4.93	6.92 ± 4.81	5.29 ± 2.67	2.75 ± 0.54
IL‐17A	18.89 ± 3.43	16.28 ± 4.70	115.23 ± 214.57	48.79 ± 64.15	11.68 ± 4.99	13.38 ± 1.44	12.74 ± 2.84	11.66 ± 6.07
IL‐23	32.41 ± 8.78	31.79 ± 7.77	94.32 ± 103.09	219.51 ± 142.26	1388.29 ± 2539.49	43.64 ± 13.47	40.22 ± 14.93	49.75 ± 38.64
IL‐27	34.17 ± 61.71	4.94 ± 1.38	3.73 ± 0.92	4.94 ± 1.93	7.85 ± 6.70	16.69 ± 25.48	9.81 ± 10.95	2.9 ± 1.12

Abbreviations: BMSCs, bone mesenchymal stem cells; GC, glucocorticoid; H‐D, high‐dose; L‐D, low‐dose; M‐D, moderate‐dose; SLE‐C, systemic lupus erythematosus control.

**Table 3:** The levels of nine chemokines in mice.

	SLE‐C	BMSC	L‐D	M‐D	H‐D	L‐D + B	M‐D + B	H‐D + B
MCP‐1	40.12 ± 11.64	37.61 ± 13.44	31.29 ± 12.65	45.35 ± 5.67	17.24 ± 4.75	37.13 ± 22.55	54.03 ± 31.38	23.15 ± 9.76
MCP‐3	132.04 ± 12.59	118.70 ± 24.22	90.57 ± 33.85	101.59 ± 12.15	100.34 ± 22.95	113.16 ± 26.58	86.80 ± 35.37	80.35 ± 20.35
MIP‐1α	6.44 ± 5.47	2.09 ± 0.28	1.67 ± 0.72	1.92 ± 0.30	2.36 ± 1.03	3.98 ± 4.31	1.83 ± 1.08	1.39 ± 0.43
MIP‐1β	3.96 ± 2.41	4.98 ± 1.44	5.82 ± 5.31	4.06 ± 1.67	2.97 ± 1.47	3.54 ± 1.53	3.22 ± 1.84	1.99 ± 0.24
MIP‐2	7.86 ± 1.98	7.93 ± 1.96	6.21 ± 0.76	4.84 ± 2.45	4.23 ± 1.84	6.30 ± 1.23	7.62 ± 2.90	3.84 ± 1.17
Eotaxin	84.05 ± 16.67	67.64 ± 11.87	67.82 ± 23.07	72.52 ± 19.82	113.11 ± 62.12	70.37 ± 23.85	49.43 ± 20.06	7.94 ± 13.86
RANTES	97.8 ± 31.20	85.16 ± 24.03	36.77 ± 11.3	46.96 ± 8.74	33.86 ± 6.12	66.91 ± 25.90	34.79 ± 15.09	32.15 ± 6.62
GRO‐α	19.01 ± 8.94	13.55 ± 3.89	7.49 ± 2.79	44.47 ± 67.73	5.87 ± 2.87	5.22 ± 5.77	11.03 ± 6.91	10.64 ± 16.50
IP‐10	66.96 ± 11.004	62.37 ± 4.78	40.25 ± 6.81	43.57 ± 9.01	35.60 ± 4.90	45.43 ± 11.75	33.55 ± 12.94	34.42 ± 4.88

Abbreviations: BMSCs, bone mesenchymal stem cells; GC, glucocorticoid; H‐D, high‐dose; L‐D, low‐dose; MCP, monocyte chemoattractant protein; M‐D, moderate‐dose; MIP, macrophage inflammmatory protein; SLE‐C, systemic lupus erythematosus control.

## 4 DISCUSSION

Latest advancements in osteoimmunology have brought to light the critical influences of imbalances within the microenvironment and disruptions in immune regulation on the initiation and progression of osteoporosis.^21^, ^22^, ^23^ Maintaining bone mass relies on the intricately coordinated process of bone remodeling, necessitating harmonious interactions among various cell types within the bone marrow microenvironment. This orchestration includes achieving a delicate equilibrium between bone formation and bone resorption, facilitated by an array of cytokines. Perturbations in pertinent factors and signaling pathways within the bone marrow microenvironment can lead to an imbalance in bone metabolism, ultimately culminating in osteoporosis.^24^ Although bisphosphonates (BP) have clear efficacy in improving bone density and reducing the risk of fractures, safety concerns such as atypical femoral fractures, osteonecrosis of the jaw, atrial fibrillation, and gastrointestinal intolerance have become increasingly recognized with clinical experience. Additionally, BP should be avoided in patients with severe renal impairment, during pregnancy, and lactation.^25^ As MSCs with their multipotent capabilities have found extensive applications in the realm of regenerative medicine in the past decades,^26^ exogenous MSCs through transplantation is anticipated to rectify immune microenvironment imbalances and offer a means to treat osteoporosis.

It is becoming increasingly evident that in patients with SLE who also have osteoporosis, the immune‐inflammatory responses of the underlying disease play significant roles in affecting bone metabolism, alongside the use of GCs.^27^, ^28^ SLE itself can contribute to bone loss, degradation of cortical microarchitecture, and reduced bone strength, even in the absence of GC treatment.^29^, ^30^ However, GCs are a notable factor in causing secondary osteoporosis in SLE patients.^31^ By binding to the GC receptor, their interaction can lead to the extension of osteoclast lifespan, stimulation of osteoblast and osteocyte apoptosis, acceleration of the breakdown of 25‐hydroxyvitamin D and 1, 25‐dihydroxyvitamin D, as well as decreased calcium absorption in the gastrointestinal tract and reduced calcium reabsorption in renal tubules.^30^ Alendronate, a BP, has been recommended in the prevention of GIOP in premenopausal patients with SLE. However, the efficacy and safety of alendronate in preventing GIOP remains controversial.^32^, ^33^ A wealth of clinical studies have substantiated that MSCs hold promise in treating SLE through diverse mechanisms, including the regulation of B cells, T cells, macrophages, and cell homing.^34^, ^35^, ^36^ The interplay between the immune effects of MSCs and their impact on secondary osteoporosis when used in conjunction with GCs for SLE treatment remains a complex matter warranting further investigation. In this study, different dosages of GC treatment were employed to systematically compare alterations in bone metabolism and the associated immune microenvironment, both with and without the application of BMSCs, using a spontaneous animal model of SLE. Our findings demonstrated that BMSCs could effectively alleviate secondary osteoporosis resulting from both the intrinsic effects of SLE and SLE coupled with diverse levels of GC therapy.

The bone structure encompasses external cortical bone and inner cancellous bone. The osteon‐dense bone, also known as cortical bone, is made up of compact osteons arranged in an organized manner, while the cancellous bone consists of trabecular bones. Although BMSC treatment exhibited no differences in trabecular and cortical bone mineral density (Tb.BMD and Ct.BMD) among various groups, it could significantly increase the mineral content (BMC) of both trabecular and cortical bones, with this effect being more pronounced in cancellous bones. BMSC treatment also exhibited statistically significant improvements in trabecular tissue volume (Tb.TV), trabecular bone volume (Tb.BV), trabecular bone volume fraction (Tb.BV/TV) and trabecular bone surface area/bone volume ratio (Tb.BS/BV). BV/TV is a commonly used index for evaluating bone mass. For trabecular bone, this ratio reflects the amount of trabecular bone mass, with an increase indicating that bone synthesis metabolism exceeds resorption. Our results demonstrated that GC treatment promoted bone resorption, while BMSC treatment can reverse this process. The higher the GC dose, the more significant the therapeutic effect of BMSC becomes. These findings suggest that BMSC treatment significantly amplifies both bone formation and mass, leading to improvements in bone metabolism.

Bone quality is closely related to the trabecular microarchitecture. BMSC treatment did not improve the trabecular number (Tb.N), but had a positive effect on trabecular thickness (Tb.Th). Additionally, BMSC treatment yielded improvements in structural aspects such as the SMI, connectivity, and FD of trabecular bone. In our study, the improvement of trabecular structure with BMSC treatment was primarily attributed to its increase in trabecular number. In cortical bones, BMSC significantly increased total area (Tt.Ar) and bone area (Ct.Ar), however, this effect was not based on improvement in cotical thickness (Ct.Th). Altogether, our results underscore that BMSC treatment contributes to heightened mineral content in bone and enhanced architectural integrity in trabecular bone structures.

GIOP is a recognized adverse drug reaction resulting from the use of GCs.^37^, ^38^ Interestingly, the changes in favor of osteoporosis were not consistently observed following GC treatment. In some cases, a tendency to improved osteoporosis was observed in our SLE models. For instance, various therapeutic doses of GC led to enhancements in Tb.BV/TV; however, this improvement was negated upon the addition of BMSCs. It can be inferred that multiple factors influence bone metabolism in SLE‐related osteoporosis. GCs might enhance specific indicators by modulating the immune inflammation associated with the disease itself, while also triggering the development of GIOP. It's important to note that the osteoporosis occurring in SLE during GC treatment cannot be solely explained by GIOP. Previous studies on murine osteoporosis models have revealed that the effects of MSC treatment from different sources for diverse causes of osteoporosis can vary in terms of their impact on osteoporosis‐related indicators. Human umbilical cord blood MSCs can reverse osteoporosis in non‐obese diabetic mice/severe combined immunodeficency mice mice by altering osteoblastic and osteoclastic activities.^39^ Tonsil‐derived MSCs simultaneously promote bone mineralization, enhance osteogenic differention and recover osteoporotic bone mass.^40^, ^41^ Similarly, in ovariectomized mice‐induced osteoporotic mouse model, the application of human‐derived MSCs significantly reduced trabecular bone loss in the distal femoral metaphysis.^42^ The effectiveness of ip MSC injection has been validated. A study reported that both ip and intra‐articular (ia) MSC injection resulted in a beneficial clinical and histological effect on proteoglycan induced arthritis mouse model.^43^ In this study, we demonstrated that ip injection of mouse‐derived MSCs led to augmented bone mass, improved bone structure, and facilitated bone formation. Furthermore, these improvements appeared to stem from more intricate pathological mechanisms beyond depending on the RANK/RANKL and OPG pathways entirely, suggesting that the onset and progression of osteoporosis might involve multifaceted factors.

The interventions of MSC and GC have significant impacst on the immune system. BMSC and GC treatments reduced the number of B cells in the bone marrows of mice, but the effect was not significant in the spleens. While memory B cells in the spleen were significantly enhanced in the moderate and H‐D GC intervention groups, the corresponding BMSC treatment groups not. This suggested that B cells in the spleen might not play key roles in the therapeutic effects of BMSCs. The reduction in bone marrow B cell numbers hints at the possibility that osteoporosis‐associated autoantibodies in the bone marrow contribute to osteoporosis through a direct immune response.

Regarding T cell subsets and associated cytokines, BMSCs significantly promote the expression of IL‐10, thereby indirectly exerting an inhibitory effect on Th1 differentiation. This finding aligns with previous studies indicating that IL‐10 plays a crucial role in MSC homing and bone regeneration after fractures.^44^, ^45^ Patricia et al. reported that MSCs could generate a regulatory T cell population during the differentiation process of Th1 and Th17 cells.^46^ Similarly, our results demonstrated that BMSCs exert immunomodulatory effects by reducing Th17 expression and rectifying the Th17/Treg imbalance. It's noteworthy that different doses of GCs exhibit different effects when combined with BMSC interventions. Although there were no differences in the population of Treg cells in the BMSC, H‐D, or M‐D groups, there was a dramatic decrease with H‐D dexamethasone treatment, and this decline was not ameliorated by the addition of BMSCs. In contrast, Th17 was significantly reduced in the BMSC and H‐D groups, but not in the L‐D and M‐D groups. After adding BMSCs, although not statistically significant, we observed a decrease in Th17 expression in these two groups. These results might suggest that H‐D GC treatment exerts a potent anti‐inflammatory effect but may inhibit the therapeutic potential of BMSCs in various aspects of immune regulation. The immunomodulatory effect of MSCs appears to diminish after M‐D to H‐D hormone therapy, which could have important implications for clinical applications.

## 5 CONCLUSIONS

In summary, our study investigated the effects of BMSCs and different doses of GC interventions on osteoporosis in SLE murine models. Our findings demonstrated that BMSCs could effectively alleviate osteoporosis induced by SLE itself, as well as osteoporosis resulting from SLE combined with various doses of GC therapy. BMSCs achieve this by enhancing bone mass, improving bone structure, and promoting bone formation through increased bone mineral content and optimization of trabecular morphology. These therapeutic effects of BMSCs appear to be mediated by their influence on bone marrow B cells, T cell subsets, and associated cytokines. It is worth noting that H‐D GC treatment exerts a potent anti‐inflammatory effect but may hinder the immunotherapeutic potential of BMSCs. Therefore, our research may offer valuable guidance to clinicians regarding the use of BMSC treatment in SLE and provide insights into the judicious use of GCs in clinical practice.

## AUTHOR CONTRIBUTIONS

**Sisi Ding**: Funding acquisition; investigation; writing—original draft. **Tian Ren**: Investigation; resources. **Saizhe Song**: Investigation; methodology. **Cheng Peng**: Investigation; methodology. **Cuiping Liu**: Funding acquisition; supervision. **Jian Wu**: Conceptualization; supervision; writing—review and editing. **Xin Chang**: Conceptualization; funding acquisition; project administration; writing—review and editing.

## CONFLICT OF INTEREST STATEMENT

The authors declare no conflict of interest.

## ETHICS STATEMENT

All procedures performed in studies involving were reviewed and approved by the Ethics Review Board of the First Affiliated Hospital of Soochow University (Ethical No.2020105) in accordance with the Helsinki Declaration of 1975, as revised in 2008. Consented patients were informed of the use of their clinical information before sample collection. The authors confirm that they have obtained written consent from each patient to publish the manuscript.

## Data Availability

The data sets generated and analyzed during the current study are available from the corresponding author on reasonable request.
